# ALOX5‐5‐HETE promotes gastric cancer growth and alleviates chemotherapy toxicity via MEK/ERK activation

**DOI:** 10.1002/cam4.4066

**Published:** 2021-06-13

**Authors:** Jianjun Tang, Chuang Zhang, Jingjing Lin, Peng Duan, Jian Long, Hongyan Zhu

**Affiliations:** ^1^ Department of General Surgery Xiangyang No.1 People’s Hospital Hubei University of Medicine Xiangyang China; ^2^ Department of Pediatrics Xiangyang No.1 People’s Hospital Hubei University of Medicine Xiangyang China; ^3^ Department of Blood Transfusion Xiangyang Traditional Chinese Medicine Hospital Xiangyang China; ^4^ Department of Obstetrics and Gynaecology Xiangyang No.1 People’s Hospital Hubei University of Medicine Xiangyang China; ^5^ Department of Oncology Jingzhou Central Hospital The Second Clinical Medical College Yangtze University Jingzhou China; ^6^ Department of Oncology Xiangyang No.1 People’s Hospital Hubei University of Medicine Xiangyang China

**Keywords:** 5‐HETE, Alox5, chemotherapy, gastric cancer, MEK/ERK, zileuton

## Abstract

**Background:**

Recent studies highlight the regulatory role of arachidonate lipoxygenase5 (Alox5) and its metabolite 5‐hydroxyeicosatetraenoic acid (5‐HETE) in cancer tumorigenesis and progression. In this study, we analyzed the expression, biological function and the downstream signaling of Alox5 in gastric cancer.

**Methods:**

Alox5 protein levels were measured using IHC and ELISA. Growth, migration and survival assays were performed. Phosphorylation of molecules involved in growth and survival signaling were analyzed by WB. Analysis of variance and *t*‐test were used for statistic analysis.

**Results:**

Alox5 and 5‐HETE levels were upregulated in gastric cancer patients. ALOX5 overexpression or 5‐HETE addition activates gastric cancer cells and reduces chemotherapy’s efficacy. In contrast, ALOX5 inhibition via genetic and pharmacological approaches suppresses gastric cancer cells and enhances chemotherapy’s efficacy. In addition, Alox5 inhibition led to suppression of ERK‐mediated signaling pathways whereas ALOX5‐5‐HETE activates ERK‐mediated signaling in gastric cancer cells.

**Conclusions:**

Our work demonstrates the critical role of ALOX5‐5‐HETE in gastric cancer and provides pre‐clinical evidence to initialize clinical trial using zileuton in combination with chemotherapy for treating gastric cancer.

## Graphical abstract

Our work demonstrates that the ALOX5‐5‐HETE axis promotes gastric cancer growth and alleviates chemotherapy toxicity via MEK/ERK activation and provides pre‐clinical evidence to initialize clinical trials using zileuton in combination with chemotherapy for the treatment of gastric cancer.

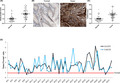

## 1 INTRODUCTION

Gastric cancer is the second most common cancer type and the third leading cause of cancer‐related mortality worldwide.^1^ There are two main histological types: intestinal type which consists of glandular solid cells and diffuse type which is composed of poorly cohesive cells. Mixed‐type consisting of intestinal‐ and diffuse‐types is found in ~25% of all gastric cancer and they display worse prognosis than non‐mixed‐type.^2^ Besides surgical resection, first‐line platinum‐ and fluoropyrimidine‐based chemotherapy is the current treatment option for gastric cancer patients with advanced disease.^3^ Unfortunately, many patients eventually experience relapse and their survival outcomes are poor.^4^ The mechanism of gastric cancer pathogenesis and development is still largely unknown.

Chronic inflammation is known to be one of the most essential etiological factors in gastrointestinal cancer.^5^ Arachidonate lipoxygenase5 (ALOX5) is a dioxygenase enzyme that catalyzes the peroxidation of arachidonic acid. It is linked to gastrointestinal cancer progression due to its central regulatory role in inflammation.^6^ Colon, esophagus, and oral cancers demonstrate the upregulation of ALOX5.^7^, ^8^, ^9^ Serum ALOX5 is elevated in breast cancer patients and can serve as a progressive protein marker.^10^ Alox5 metabolizes arachidonic acid to 5‐hydroxyeicosatetraenoic acid (5‐HETE).^11^ Alox5‐5‐HETE confer growth, invasion, and chemopreventive advantage in cancer cells.^9^, ^12^ Alox5 activation is required for nicotine‐mediated epithelial‐mesenchymal transition and gastric cancer cell growth.^13^ However, the expression, biological function, and downstream signaling mechanism of ALOX5 in gastric cancer have not been systematically evaluated.

In this work, we analyzed the levels of ALOX5 and 5‐HETE on tumor tissues and corresponding normal counterparts from gastric cancer patients using quantitative and qualitative methods. We performed functional and mechanism analyses on multiple gastric cancer cell lines using gain‐of‐function and loss‐of‐function approaches. Our results demonstrate that the ALOX5‐5‐HETE axis promotes gastric cancer growth and alleviates chemotherapy toxicity via MEK/ERK activation.

## 2 MATERIALS AND METHODS

### 2.1 Patient tissue specimens, cell lines, and compounds

Patient tissue specimens were obtained from patients seen at Xiangyang No.1 People's Hospital and Jingzhou Central Hospital after signed informed consent as approved by Xiangyang No.1 People's Hospital Institutional Review Board and the Yangtze University Centralized Institutional Review Board. Gastric tumor and adjacent normal gastric tissues (at least 3 cm away from tumor) were removed during surgery. Patients’ clinicopathological features are presented in Supplementary Table S1. The patient inclusion criteria used in the study are for all gastric cancer patients eligible for surgery. Gastric cancer cell lines N87 and AGS were obtained from the Cell Bank of Shanghai Institute of Biological Science. Cell lines were authenticated using a 9‐short tandem repeat (STR) profile analysis. Cells were cultured in MEM medium supplemented with 10% FBS, 2 mM L‐glutamine, and 1% of penicillin‐streptomycin (Invitrogen) in a humidified incubator at 37℃ with 5% CO_2_. Fluorouracil (5‐FU), cisplatin, LY294002, and U0126 were obtained from Selleckchem. Alox5 inhibitors zileuton and AA861 were obtained from Cayman Chemical. All compounds were reconstituted following the manufacturer's instructions and stored as frozen stocks at −20℃.

### 2.2 Immunohistochemistry

Immunohistochemistry was performed on paraffin‐embedded tissues. The blocks were sliced into 5‐μM thick sections, deparaffinized with xylene, and rehydrated with decreasing concentrations of ethanol in water. Slides were boiled in citric buffer for antigen retrieval and then fixed with 4% paraformaldehyde. Slides were incubated with 1:50 diluted anti‐Alox5 antibody (PA5‐78762; Thermo Fisher Scientific) for 2 h at room temperature. After rinsing slides in PBS, slides were incubated with secondary antibody (1:100 biotinylated goat anti‐rabbit IgG) for 45 min at room temperature. After washing with PBS, sections were stained with diaminobenzidine tetrahydrochloride (DAB) solution (Vector Laboratories). The sections were counterstained with hematoxylin solution (Sigma) and sealed with a cover‐slip using a mounting medium (Thermo Fisher Scientific).

### 2.3 5‐HETE and Alox5 measurement

Tissue or cellular 5‐HETE was quantified using the 5‐HETE ELISA kit (Abcam). Tissue or cellular Alox5 was quantified using the Alox5 ELISA kit (Aviva Systems Biology). Tissue homogenates and cell lysates were prepared according to the manufacturer's instructions. The samples were adjusted to 20 ng/ml of concentration using solution buffer provided in the kit and added to microplates coated with antibodies against 5‐HETE or Alox5. Proteins were quantified calorimetrically using a Microplate Reader (SpectraMax).

### 2.4 Cellular assays

Cell proliferation was measured using BrdU Cell Proliferation ELISA Assay Kit (Cell Signaling). Cell apoptosis was assessed by measuring cytosolic oligonucleosome‐bound DNA using a Cell Death ELISA kit (Roche). The absorbance at 450 nm was measured on a microplate reader (SpectraMax). Migration was assessed using Boyden Chamber assay (Cell Biolabs). In brief, cells were seeded on the topside of the insert in serum‐free media and a medium with 10% serum was placed in the well below. After 6–8 h incubation in a cell culture incubator, migratory cells moved through the pores toward the serum below. These were then were fixed with 10% formalin, stained with Giemsa, and counted under a light microscope.

### 2.5 Western blotting

Whole‐cell proteins were extracted using RIPA buffer containing protease inhibitor cocktails and phosphatase inhibitor (Life Technologies). Proteins were fractionated by SDS‐PAGE and transferred to a PVDF membrane. After incubation with 5% BSA in TBST for 1h, the membrane was incubated with antibodies against p‐ERK(T202/Y204, 1:1000), ERK (1:2000), p‐p90RSK(1:1000), p90RSK(1:2000), p‐Akt(S473, 1:1000), Akt(1:2000), pol II S5 (1:1000), pol II(1:2000), Bim (1:2000), Mcl‐1(1:1000), Bcl‐2(1:1000), and β‐actin (1:3000) at 4℃ overnight. Antibodies were obtained from Santa Cruz Inc. Membranes were washed three times for 10 min and incubated with a 1:3000 dilution of horseradish peroxidase‐conjugated anti‐mouse or anti‐rabbit antibodies for 2 h. Blots were developed with the ECL system (Amersham Biosciences).

### 2.6 Cell transfection

To overexpress human ALOX5 or achieve specific knockdown of ALOX5, 10^5^ cells per well were seeded in 6‐well plates. The next day, cells were incubated for 24 h in serum‐free IMEM containing a mixture of 1 μg p‐ALOX5 plasmid DNA or 100 ng siRNA and TurboFect transfection reagent (Fermentas GmbH). Control cells were transfected with an empty pcDNA vector or scramble siRNA plus TurboFect. ALOX5‐overexpressing plasmid (pCMV6‐ALOX5, RG217259) and vector control plasmid (pCMV6‐AC, PS100010) were obtained from OriGene. Two specific siRNA targeting ALOX5 (ALOX5 siRNAa and siRNAb) and a non‐specific scrambled siRNA (Scr siRNA) were synthesized by GenePharma Inc.

### 2.7 Statistical analyses

**FIGURE 1:**
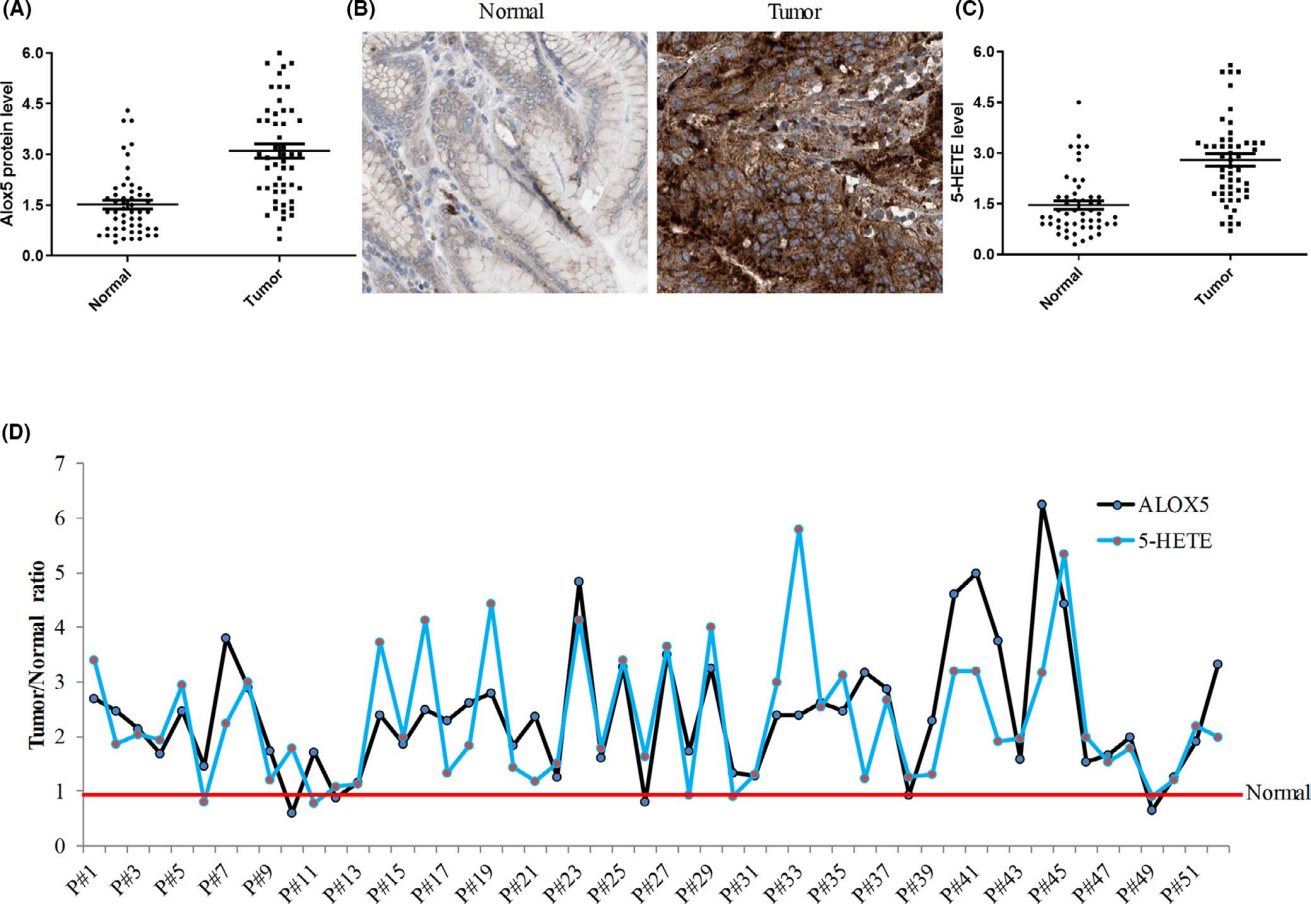
Alox5‐5‐HETE axis is upregulated in gastric cancer tissues. (A) Scatter plot of Alox5 protein level in paired normal and tumor tissues obtained from 52 patients with gastric tumor. (B) Representative immunohistochemistry analysis of normal and tumor gastric tissues from patient#23 performed by Alox5 staining. (C) Scatter plot of 5‐HETE level in paired normal and tumor tissues obtained from 52 patients with gastric tumor. Alox5 and 5‐HETE were assessed using tissue homogenates and quantified using ELISA assay. (D) Relative tumor/normal ratio value of Alox5 and 5‐HETE levels in individual gastric cancer patients. Alox5 or 5‐HETE level in normal tissues were set as 1 (indicated by a red line)

**FIGURE 2:**
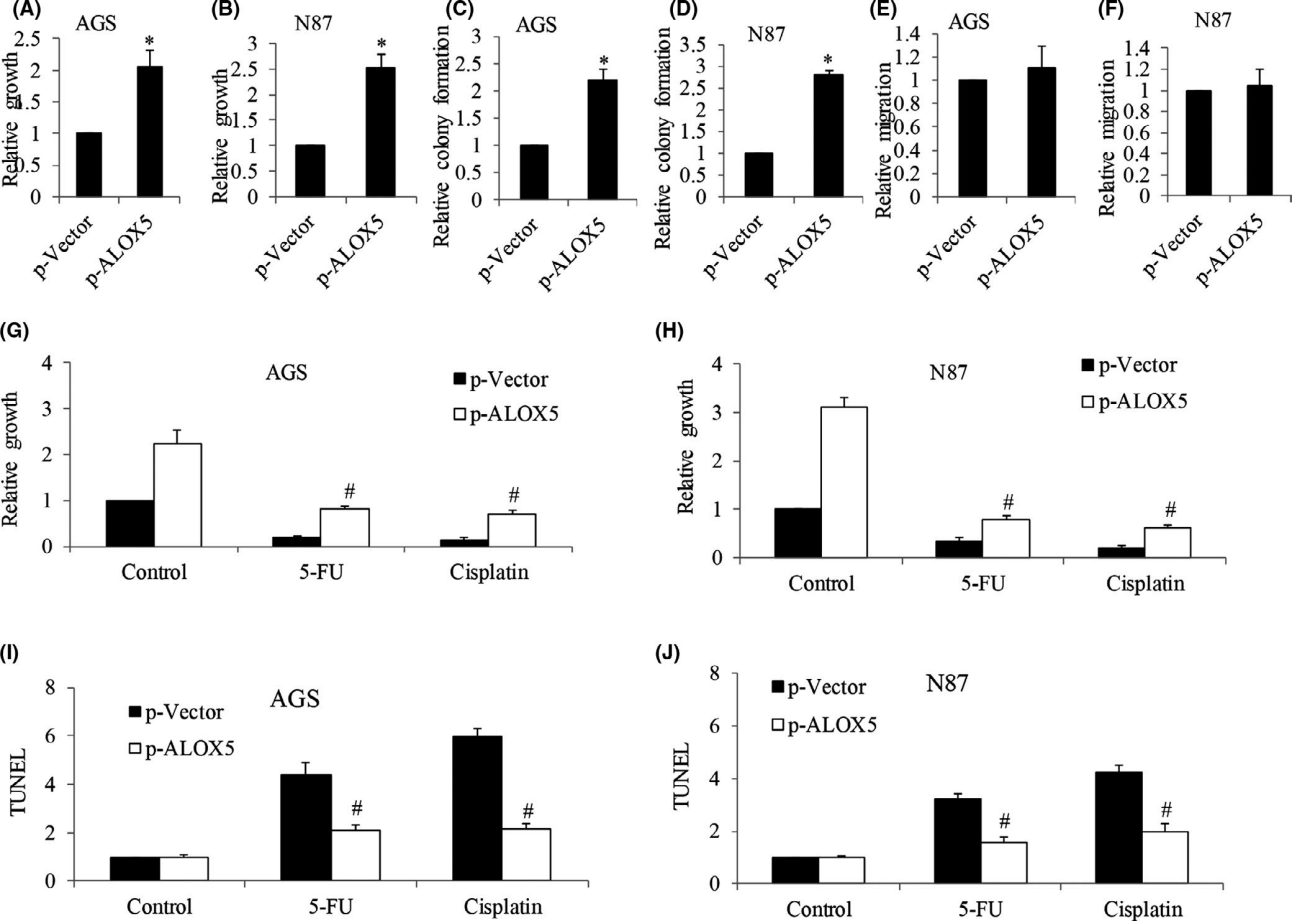
ALOX5 overexpression promotes cell growth and protects gastric cancer cells from chemotherapeutic agents‐induced toxicity. (A and B) Analysis of proliferation of AGS and N87 cells after ALOX5 overexpression performed by BrdU labeling. (C and D) Analysis of colony formation of AGS and N87 cells after ALOX5 overexpression. (E and F) Analysis of migration of AGS and N87 cells after ALOX5 overexpression. Analysis of proliferation (G and H) and apoptosis (I and J) after treatment of 5‐FU and cisplatin in ALOX5‐overexpressing AGS and N87 cells. Results are presented as relative to control. Proliferation and apoptosis assays were assessed after 72 h of drug treatment. 5‐FU at 200 nM and cisplatin at 300 nM were used. *p < 0.05, compared to p‐Vector. #*p *< 0.05, compared to cisplatin or 5‐FU alone

**FIGURE 3:**
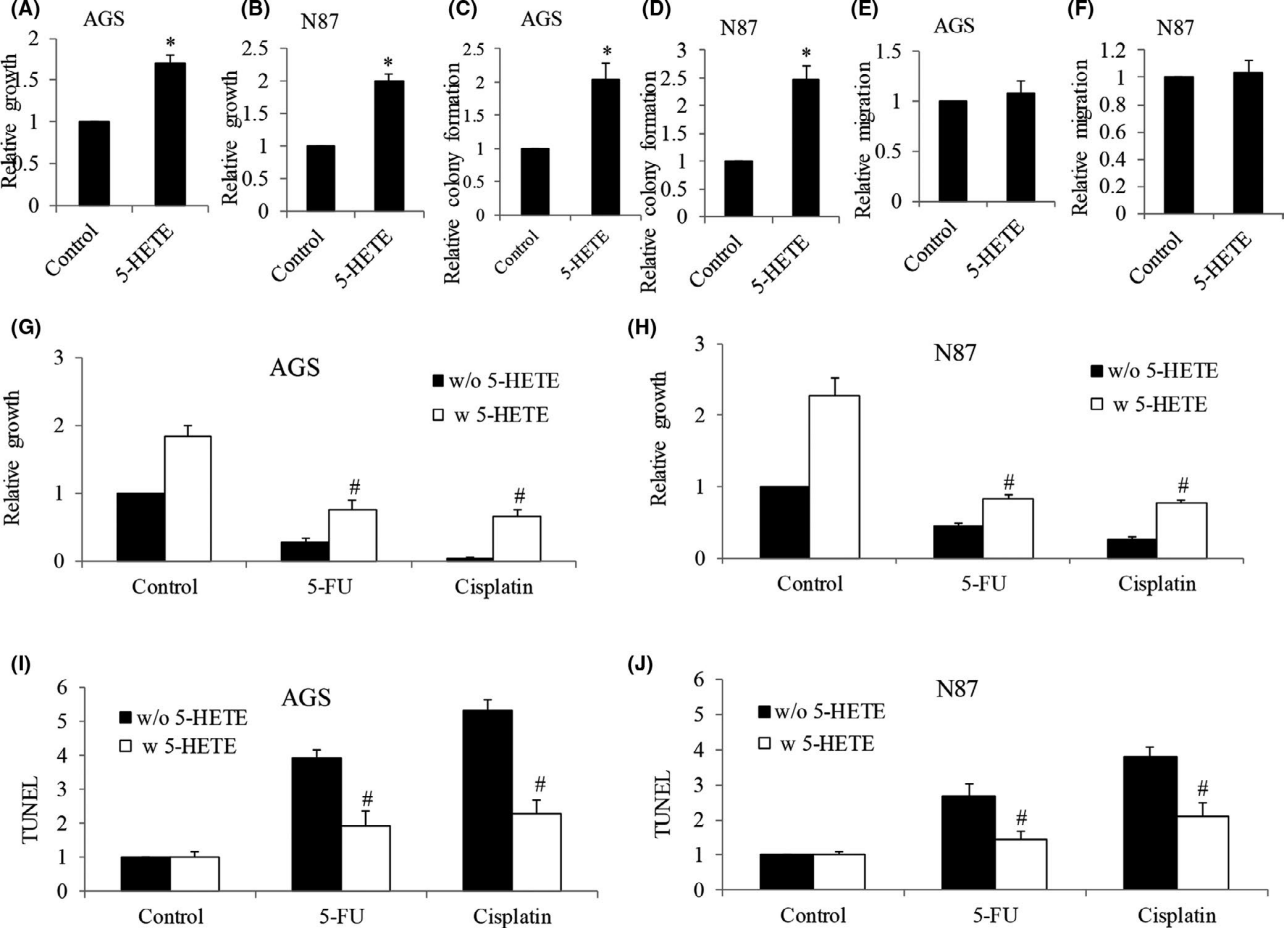
Addition of 5‐HETE promotes cell growth and protects gastric cancer cells from chemotherapeutic agents‐induced toxicity. (A and B) Analysis of proliferation of AGS and N87 cells after 5‐HETE treatment, as evaluated by BrdU labeling. (C and D) Analysis of colony formation of AGS and N87 cells after 5‐HETE treatment. (E and F) Analysis of migration of AGS and N87 cells after 5‐HETE treatment. Analysis of proliferation (G and H) and apoptosis (I and J) after treatment of 5‐FU and cisplatin in the presence of 5‐HETE in AGS and N87 cells. Results are presented as relative to control. Proliferation and apoptosis assays were assessed after 24 h of drug treatment. 5‐HETE at 500 nM, 5‐FU at 200 nM, and cisplatin at 300 nM were used. *p < 0.05, compared to p‐Vector. #*p *< 0.05, compared to cisplatin or 5‐FU alone

**FIGURE 4:**
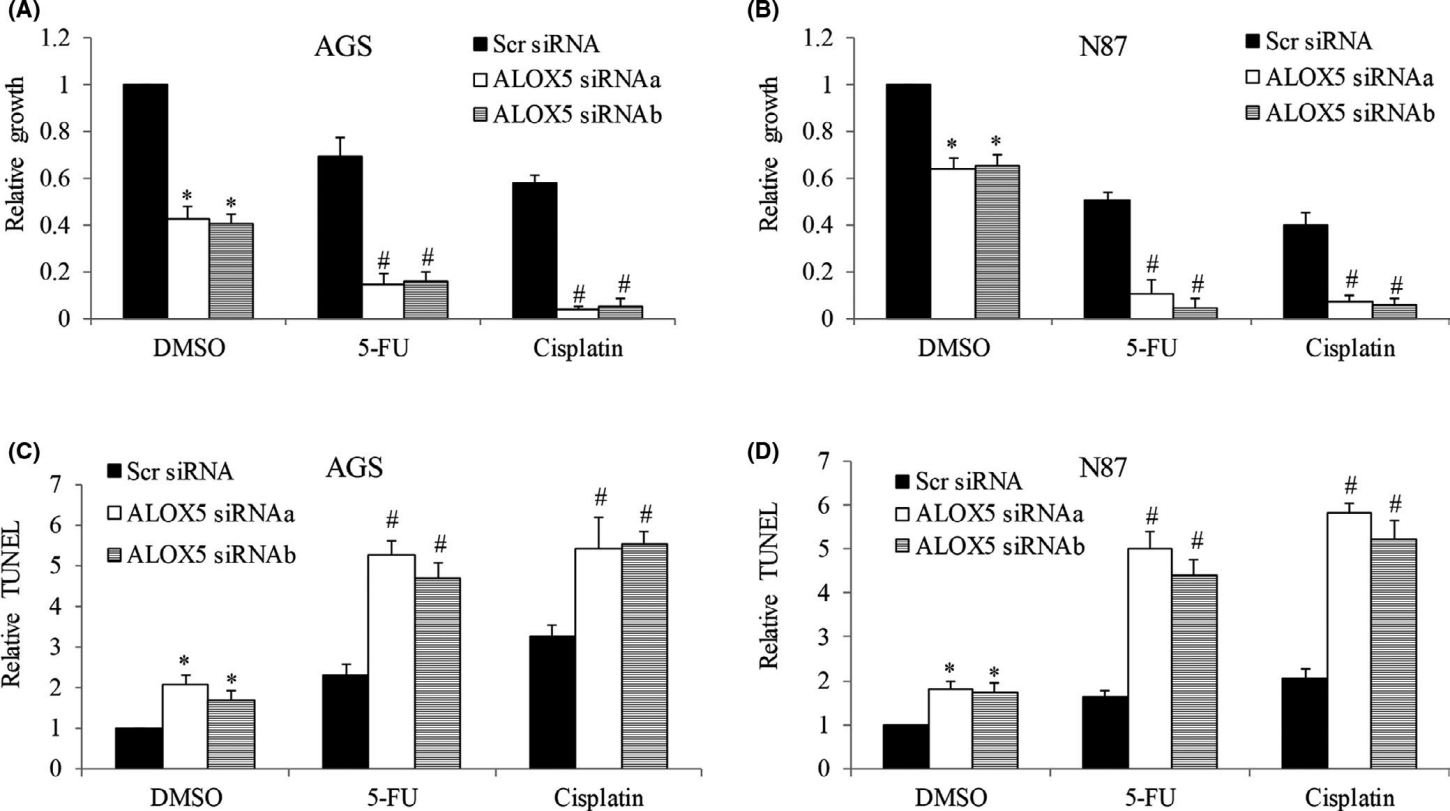
ALOX5 knockdown inhibits gastric cancer and enhances the toxicity of chemotherapeutic agents. Analysis of proliferation (A and B) and apoptosis (C and D) in AGS and N87 cells after ALOX5 knockdown, and in the presence of 5‐FU and cisplatin. ALOX5 knockdown significantly enhances the anti‐proliferative and pro‐apoptotic effects of 5‐FU and cisplatin in gastric cancer cells. 5‐FU at 50 nM and cisplatin at 50 nM were used. Proliferation and apoptosis assays were assessed after 72 h of drug treatment. Results are presented as relative to control. *p < 0.05, compared to control. #*p *< 0.05, compared to cisplatin or 5‐FU

**FIGURE 5:**
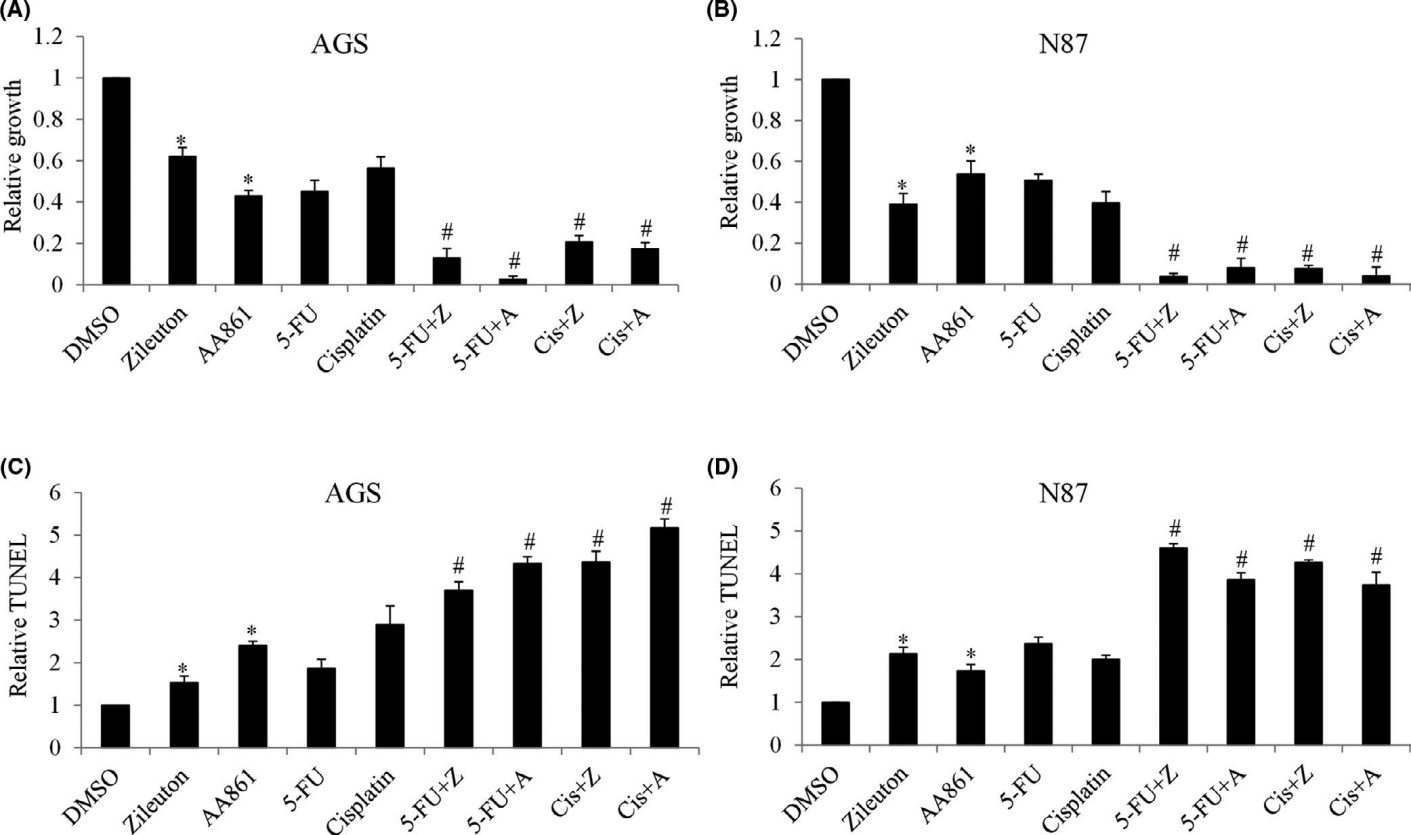
Alox5 inhibitors suppress gastric cancer and enhance the toxicity of chemotherapeutic agents. (A and B) Proliferation level of AGS and N87 cells after zileuton and AA861 treatment in the presence of 5‐FU and cisplatin, as evaluated by BrdU labeling. (C and D) Proliferation level of AGS and N87 cells after zileuton and AA861 treatment in the presence of 5‐FU and cisplatin, as evaluated by TUNEL assay. Zileuton and AA861 significantly enhance the anti‐proliferative and pro‐apoptotic effects of 5‐FU and cisplatin in gastric cancer cells. 5‐FU at 50 nM and cisplatin at 50 nM were used. Zileuton at 100 μM and 200 μM, AA86 at 30 μM and 60 μM were used in proliferation and apoptosis assays, respectively. Proliferation and apoptosis assays were assessed after 72 h of drug treatment. *p < 0.05, compared to control. #*p *< 0.05, compared to cisplatin or 5‐FU

Data are expressed as mean and standard deviation (SD) to indicate data variability. For Figure 1, a one‐way analysis of variance (ANOVA) and post hoc Tukey honestly significant difference (HSD) test were used. Each data was compared with normalized normal result and all analyses were performed using SPSS software. Correlation analysis among Alox5 and 5‐HETE was performed using Spearman Rank Correlation Analysis. Analysis was conducted using PRISM 8.0 software. A two‐tailed unpaired *t*‐test was used for Figures 2, 3, 4, 5. These results were obtained from at least three independent experiments. A *p*‐value <0.05 was considered statistically significant.

## 3 RESULTS

### 3.1 Alox5‐5‐HETE axis is activated in gastric cancer patients

To investigate if differences in Alox5 protein levels between normal and tumor gastric tissues are significant, we performed both ELISA (quantitative) and IHC (qualitative) analyses. This uses paired normal and tumor tissues obtained from gastric cancer patients during surgery. The clinicopathological characteristics of 36 patients diagnosed with gastric cancer are summarized in Supplementary Table S1, including age, TNM stage, tumor sites, and histology. Scatter plot from ELISA analysis showed that the average Alox5 protein level is upregulated in gastric cancer tissues compared to their corresponding normal counterparts (Figure 1A), and furthermore, the upregulation is regardless of patients’ clinicopathological features. Representative images of IHC analysis displayed the cytoplasmic/membranous staining of Alox5 and that the staining intensity was higher in tumor than normal samples (Figure 1B). We next assessed the 5‐HETE level to determine whether Alox5 was active in gastric cancer. The pattern of 5‐HETE level in normal and tumor was similar to ALOX5 as shown by the scatter plot (Figure 1C). Analysis of individual patient's tumor/normal ratio of Alox5 and 5‐HETE showed that all patients who displayed significant upregulation of ALOX5 also demonstrated higher level of 5‐HETE, except patients #6, #10, #11, #21, #26, #36, and #49 (Figure 1D). We performed a Spearman rank correlation analysis. The Spearman's correlation coefficient determined was 0.713 with *p* value less than 0.0001. This confirmed a positive correlation among Alox5 and 5‐HETE. Altogether, our results demonstrate that the Alox5‐5‐HETE signaling axis is upregulated and activated in gastric cancer.

### 3.2 Alox5‐5‐HETE axis activation alleviates toxicity induced by chemotherapeutic agents in gastric cancer

To investigate the possible role of Alox5 in gastric cancer, we performed gain‐of‐function analysis by overexpressing ALOX5 in N87 and AGS. The AGS cell line was derived from fragments of a tumor resected from a gastric cancer patient who had received no prior therapy. The N87 cell line was derived from the metastatic site of gastric cancer. Based on gene profiling cluster analysis, these two human gastric cancer cell lines adopt a different differentiation program and may possess distinct biological and genetic properties.^14^ N87 and AGS were selected to demonstrate the biological role of ALOX5 because (a) these are commonly used gastric cancer cell lines for in vitro gastric cancer model; and (b) they represent different cellular origins and genetic profiles. We first confirmed the successful overexpression by showing a 3‐ to 4‐fold increase in Alox5 protein levels in ALOX5‐overexpressing AGS and N87 cells (Figure S1A and S1B). As expected, we observed an increase in the 5‐HETE level (Figure S1C and S1D). Functional analysis showed that ALOX5 overexpression increased gastric cancer proliferation as well as colony formation by 2‐ to 3‐fold (Figure 2A–2D). In contrast, ALOX5 overexpression did not affect gastric cancer migration (Figure 2E and 2F). Furthermore, ALOX5 overexpression significantly reversed the anti‐proliferative and pro‐apoptotic effects of cisplatin and 5‐FU in both N87 and AGS cells (Figure 2G–2J). It is worth noting that the increased growth and reduced responsiveness to chemotherapeutic agents are not limited to ALOX5 overexpression but also to the addition of 5‐HETE (Figure 3). These results clearly demonstrate that ALOX5‐5‐HETE axis activation promotes gastric cancer cell growth and alleviates toxicity induced by chemotherapeutic agents. In addition, the ALOX5‐5‐HETE axis is not involved in gastric cancer cell migration.

### 3.3 Alox5 inhibition suppresses gastric cancer cells and augments chemotherapy efficacy

To confirm the role of Alox5 in gastric cancer proliferation and responsiveness to chemotherapy, we performed loss‐of‐function analysis by depleting ALOX5 using siRNA. We showed that ALOX5 depletion by ALOX5 siRNAa and siRNAb remarkably decreased Alox5 protein levels as well as 5‐HETE levels in both N87 and AGS cells (Figure S2). We further showed that ALOX5 knockdown led to 60% and 40% growth inhibition in AGS and N87 cells, respectively (Figure 4A and 4B). ALOX5 knockdown led to apoptosis in AGS and N87 cells in a similar manner (Figure 4C and 4D). To determine the effect of ALOX5 knockdown on gastric cancer cell responsiveness to chemotherapy, we exposed control and ALOX5 knockdown cells to chemotherapeutic agents at sublethal concentration. We found that compared to control (Scr siRNA) cells, ALOX5‐depleted cells were significantly more sensitive to 5‐FU and cisplatin in inhibiting growth and inducing apoptosis (Figure 4). Consistently, Alox5 specific inhibitors zileuton and 2,3,5‐trimethyl‐6‐(12‐hydroxy‐5,10‐dodecadiynyl)‐1,4‐benzoquinone (AA861), significantly inhibited proliferation and induced apoptosis of gastric cancer cells (Figure 5A and 5B). In addition, zileuton and AA861 enhanced the efficacy of 5‐FU and cisplatin in decreasing growth and survival (Figure 5C and 5D).

### 3.4 ALOX5‐5‐HETE axis stimulates gastric cancer via activating MEK/ERK pathway

**FIGURE 6:**
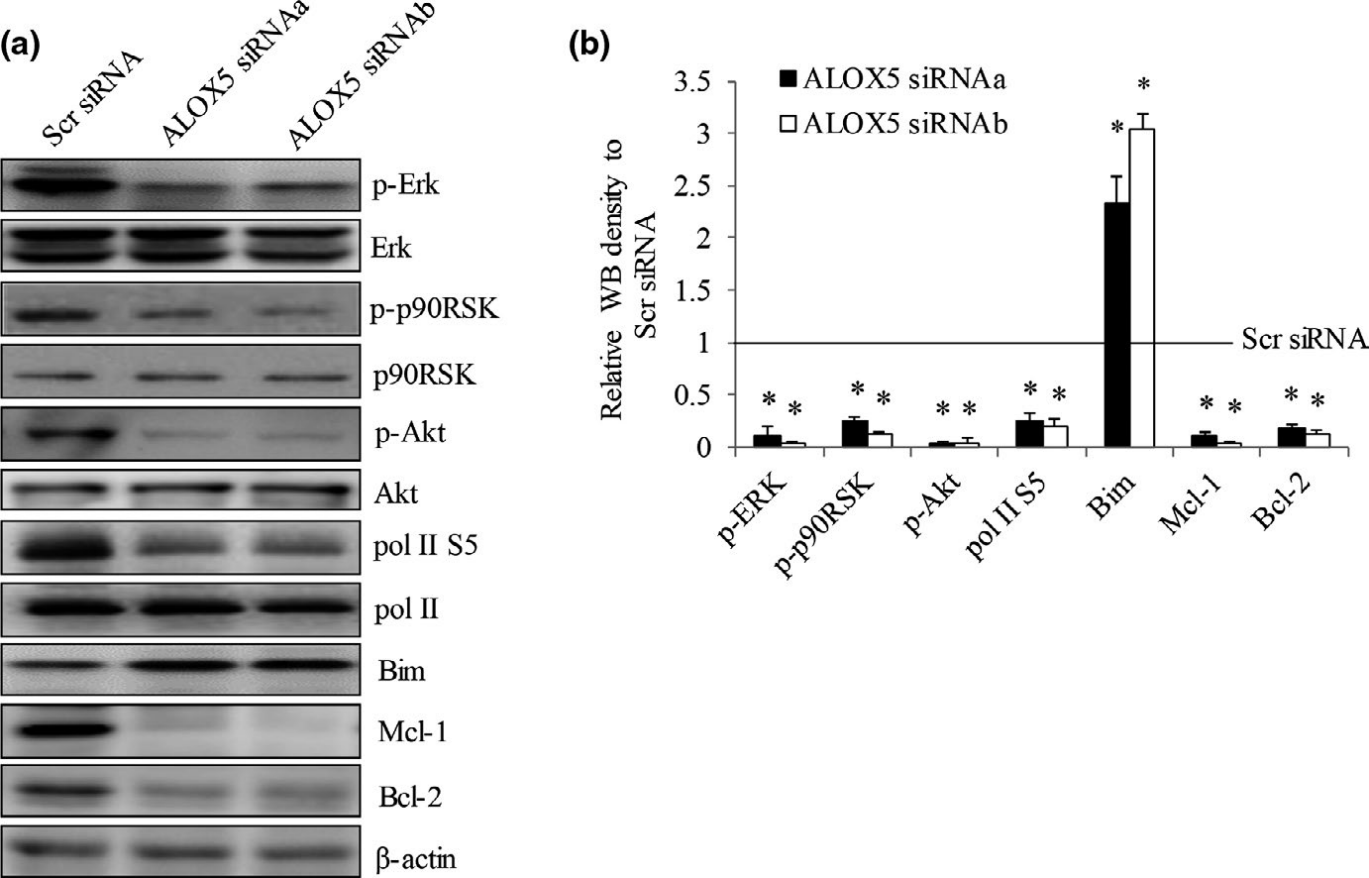
ALOX5 knockdown inhibits ERK in gastric cancer cells. Representative western blot photo (A) and quantification analysis by Image J (B) of p‐Erk, p‐p90RSK, p‐Akt, pol II S5, Mcl‐1, Bim, and Bcl levels in N87 cells after ALOX5 knockdown. Scr siRNA value was set as 1 and indicated as line. *p < 0.05, compared to Scr siRNA

To understand the downstream signaling of the ALOX5‐5‐HETE axis in gastric cancer cells, we performed western blot analysis to determine the phosphorylation status of key molecules involved in MEK/ERK and Akt pathways because these pathways play important roles in gastric cancer growth, survival, and chemoresistance.^15^, ^16^ We observed the decreased phosphorylation of ERK and its downstream effector p90RSK in ALOX5‐depleted N87 cells (Figure 6), indicating that Alox5 inhibition suppresses the MEK/ERK pathway in gastric cancer cells. We further observed the decreased phosphorylation of Akt. In addition, ALOX5 knockdown inhibited the phosphorylation of RNA polymerase II on serine 5, suggesting the suppression of transcriptional activity; and increased pro‐apoptotic protein Bim as well as decreased anti‐apoptotic proteins Mcl‐1 and Bcl‐2 in N87 cells (Figure 6).

**FIGURE 7:**
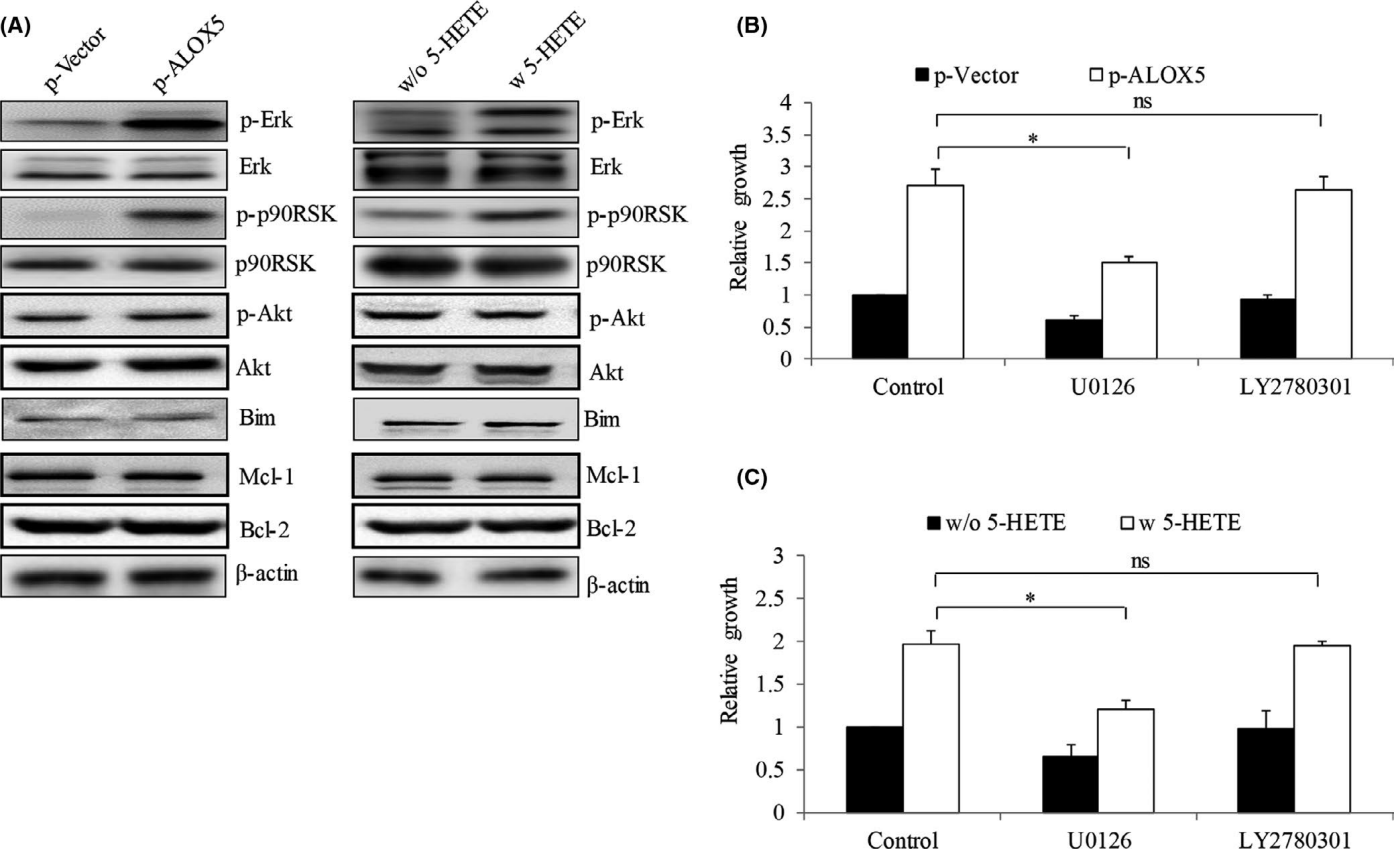
ALOX5‐5‐HETE axis activates ERK in gastric cancer cells. (A) Representative western blot photo of p‐Erk, p‐p90RSK, p‐Akt, Mcl‐1, Bim, and Bcl‐2 in N87 cells after ALOX5 overexpression or 5‐HETE addition. (B) Proliferation level of treatment of U0126 and LY2780301 in ALOX5‐overexpressing N87 cells. (C) Proliferation level of treatment of U0126 and LY2780301 in N87 cells in the presence of 5‐HETE. U0126 (MEK inhibitor, 10 μM) but not LY2780301 (Akt inhibitor, 10 μM) significantly reveres the pro‐proliferative effect induced by ALOX5 overexpression and addition of 5‐HETE. Inhibitors were added to the cells at 48h post‐transfection. *p < 0.05, compared to control; ns, not significant

In contrast, ALOX5 overexpression and addition of 5‐HETE increased p‐ERK and p‐p90RSK (Figure 7A), demonstrating the activation of ERK in gastric cancer cells. Interestingly, ALOX5 overexpression and addition of 5‐HETE did not affect levels of p‐Akt, Mcl‐1, Bcl‐2 or Bim in N87 cells (Figure 7A). We next investigated the participation of MEK/ERK and Akt in ALOX5‐5‐HETE axis‐induced proliferation using MEK inhibitor U126 and Akt inhibitor LY2780301. We showed pro‐proliferative effects of ALOX5 overexpression and 5‐HETE addition were reversed by the presence of U126 but not LY2780301 (Figure 7B and 7C). These results suggest that MEK/ERK activation is essential for ALOX5‐5‐HETE‐stimulated gastric cancer cell growth.

## 4 DISCUSSION

Gastric cancer patients suffer from poor survival outcomes and necessitate a need for molecularly targeted agents. Till now, only a few targeted therapies (e.g., trastuzumab for anti‐HER2 and ramucirumab for anti‐VEGFR2) have been developed for gastric cancer.^17^ Insights into molecular biological processes of cell signaling pathways may assist the development of new drugs and facilitate their use in drug combination trials for targeted therapies in gastric cancer. Here, our work suggests that ALOX5 is an attractive therapeutic target to sensitize gastric cancer cells to chemotherapy.

Elevated ALOX5 expression has been observed in colon cancer and esophageal adenocarcinoma.^7^, ^8^ Similarly, we found that the average Alox5 protein level was significantly higher in gastric cancer tissues compared with adjacent normal counterparts. Of note, the regulation of ALOX5 was observed in gastric cancer patients and ALOX5 expression pattern matches the 5‐HETE level. Our work extends the previous findings by suggesting that (a) ALOX5 upregulation is a common molecular feature in gastrointestinal tract cancers; (b) ALOX5 expression is heterogeneous in gastric cancer patients; and (c) activation of ALOX5‐5‐HETE axis in gastric cancer. ALOX5 expression in thyroid carcinoma is correlated with invasive tumor histopathology^12^ and serum Alox5 is a progression protein marker for breast cancer.^10^ However, we did not find any significant association between Alox5 overexpression and clinicopathological features in all 36 gastric cancer patients. The lack of association may be a result of an insufficient patient cohort, which should be further confirmed in a larger cohort study. Serum ALOX5 level in gastric patients and its association with prognosis are worthy of investigation. Gastric cancer cells display time‐dependent and dose‐dependent elevation of ALOX5 after nicotine exposure.^13^ ALOX5 is overexpressed during the process of colonic adenoma formation promoted by cigarette smoke.^18^ It is interesting to compare ALOX5 expression levels in smokers and non‐smokers with gastric cancer to understand whether ALOX5 level in gastric cancer is regulated by carcinogenic agents.

We further reveal the pro‐proliferative role of ALOX5 in gastric cancer via multiple approaches including ALOX5 overexpression, addition of 5‐HETE, genetic inhibition of ALOX5, and pharmacological inhibition of ALOX5. This is consistent with the role of ALOX5 in many other cancers.^13^, ^19^, ^20^ Although ALOX5 promotes invasion in thyroid carcinoma,^12^ ALOX5 is not critically involved in gastric cancer migration. We speculate the role of ALOX5 in cancer to be cancer type‐specific. In support of this possibility, ALOX5 has been reported to inhibit breast cancer MCF7 cell proliferation.^21^ Apart from tumor bulk cells, ALOX5 is also a critical regulator for leukemia stem cells.^22^ Our mechanism studies demonstrate that ALOX5 inhibition suppresses MEK/ERK and transcriptional activity. Consistently, ALOX5‐5‐HETE activation stimulates MEK/ERK but not Akt signaling. Although ALOX5 inhibition suppresses Akt phosphorylation, MEK inhibitor but not Akt inhibitor reverses the effects of ALOX5‐5‐HETE, demonstrating that MEK/ERK but not Akt signaling is critically involved in stimulated proliferation induces by the ALOX5‐5‐HETE axis.

As chemoresistance is the cause of treatment failure in gastric cancer patients, a significant finding of our work reveals that ALOX5 activation alleviates toxicity induced by chemotherapy. In addition, ALOX5 inhibition augments chemotherapeutic agents’ efficacy in gastric cancer. Of note, zileuton is also an FDA‐approved drug for prophylaxis and chronic treatment of allergic asthma.^23^ The anti‐cancer activities of zileuton via targeting both bulk and cancer stem cells have been recently revealed.^22^, ^24^, ^25^, ^26^ Clinical trials have been activated to examine the safety and efficacy of zileuton in combination with standard of care drugs for treating leukemia and lung cancer. Our pre‐clinical findings provide a rationale for investigating zileuton in combination with chemotherapy for gastric cancer treatment.

In conclusion, using multiple approaches, our work is the first to show that the ALOX5‐5‐HETE axis promotes gastric cancer growth and alleviates chemotherapy toxicity via MEK/ERK activation. Our findings emphasize the therapeutic value of ALOX5 inhibition in gastric cancer.

## ETHICS APPROVAL AND CONSENT TO PARTICIPATE

All procedures were approved by the institutional review board (IRB). Informed consent from patients was taken to collect clinical specimens.

## CONFLICT OF INTEREST

All authors report no conflict of interest.

## Supporting information

Supplementary Material

## Data Availability

Data are available on request from the authors.
