# Supplementary material for: ALOX5‐5‐HETE promotes gastric cancer growth and alleviates chemotherapy toxicity via MEK/ERK activation
Source: Cancer Med. 2021 Jun 13;10(15):5246–55. doi: 10.1002/cam4.4066 (PMC8335819; doi:10.1002/cam4.4066)
Supplement: Supplementary file 1 — Supplementary Material [file CAM4-10-5246-s001.doc]

**ALOX5-5-HETE axis promotes gastric cancer growth and alleviates chemotherapy toxicity via MEK/ERK activation**

**Supplemental Table 1: Clinicopathological features of gastric cancer p**atients

| **Patient Number** | Age | Tumor sites | Histology | TNM stage |
| --- | --- | --- | --- | --- |
| GC#1 | 38 | Non-cardia | Intestinal | I |
| GC#2 | 26 | other | Unknown | I |
| GC#3 | 16 | Cardia | Diffuse | III |
| GC#4 | 65 | Non-cardia | Intestinal | II |
| GC#5 | 57 | other | Intestinal | III |
| GC#6 | 48 | Cardia | Diffuse | III |
| GC#7 | 45 | Non-cardia | Mixed | II |
| GC#8 | 76 | Cardia | Unknown | I |
| GC#9 | 83 | Cardia | Intestinal | I |
| GC#10 | 75 | Non-cardia | Diffuse | III |
| GC#11 | 54 | Non-cardia | Intestinal | III |
| GC#12 | 36 | other | Diffuse | I |
| GC#13 | 64 | Cardia | Intestinal | I |
| GC#14 | 76 | Cardia | Diffuse | II |
| GC#15 | 52 | Non-cardia | Intestinal | III |
| GC#16 | 71 | Non-cardia | Unknown | III |
| GC#17 | 12 | Cardia | Diffuse | III |
| GC#18 | 53 | Non-cardia | Unknown | II |
| GC#19 | 76 | Cardia | Intestinal | II |
| GC#20 | 81 | Non-cardia | Diffuse | III |
| GC#21 | 63 | Cardia | Unknown | III |
| GC#22 | 59 | Cardia | Intestinal | I |
| GC#23 | 69 | Non-cardia | Intestinal | II |
| GC#24 | 53 | other | Diffuse | III |
| GC#25 | 59 | Non-cardia | Diffuse | II |
| GC#26 | 66 | Non-cardia | Intestinal | III |
| GC#27 | 31 | Cardia | Unknown | I |
| GC#28 | 69 | Non-cardia | Intestinal | III |
| GC#29 | 61 | Non-cardia | Intestinal | I |
| GC#30 | 63 | other | Diffuse | II |
| GC#31 | 48 | Non-cardia | Diffuse | III |
| GC#32 | 54 | Cardia | Mixed | III |
| GC#33 | 89 | Non-cardia | Diffuse | III |
| GC#34 | 34 | other | Diffuse | III |
| GC#35 | 42 | Cardia | Intestinal | II |
| GC#36 | 67 | Non-cardia | Intestinal | I |
| GC#37 | 68 | Non-cardia | Mixed | II |
| GC#38 | 76 | Cardia | Unknown | III |
| GC#39 | 54 | Cardia | Intestinal | III |
| GC#40 | 39 | Non-cardia | Diffuse | III |
| GC#41 | 76 | Non-cardia | Intestinal | II |
| GC#42 | 71 | other | Unknown | II |
| GC#43 | 69 | Cardia | Diffuse | III |
| GC#44 | 78 | Cardia | Intestinal | II |
| GC#45 | 76 | other | Unknown | I |
| GC#46 | 72 | Cardia | Intestinal | II |
| GC#47 | 65 | Non-cardia | Diffuse | III |
| GC#48 | 69 | Non-cardia | Diffuse | III |
| GC#49 | 47 | other | Intestinal | III |
| GC#50 | 49 | Non-cardia | Unknown | III |
| GC#51 | 79 | Cardia | Intestinal | II |
| GC#52 | 82 | Cardia | Unknown | II |

Tumour, node and metastasis (TNM) stage; other include Site overlapping and unspecified. Mixed is of both intestinal and diffuse.

**Fig. S1: ALOX5 overexpression activates ALOX5-5-HETE in gastric cancer.** ALOX5 overexpression increases level of ALOX5 (A and B) and 5-HETE (C and D) level in AGS and N87 cells. ALOX5 protein and 5-HETE levels were quantified using ELISA method. * p<0.05, compared to p-Vector.

**Fig. S2. ALOX5 knockdown suppresses ALOX5-5-HETE axis in gastric cancer cells.** ALOX5 overexpression remarkably decreases level of ALOX5 (A and B) and 5-HETE (C and D) level in AGS and N87 cells. ALOX5 protein and 5-HETE levels were quantified using ELISA method. * p<0.05, compared to Scr siRNA.
